# Supplementary material for: The Relation of Alpha Asymmetry to Physical Activity Duration and Intensity
Source: Brain Sci. 2025 Dec 11;15(12):1322. doi: 10.3390/brainsci15121322 (PMC12730269; doi:10.3390/brainsci15121322)
Supplement: Supplementary file 1 [file brainsci-15-01322-s001.zip › brainsci-4022144-supplementary.pdf]

Supplementary Table S1. Correspondence between the nomenclature of the present paper and variable names from GGIR outputs.

**Results file in GGIR**	**Variable names in GGIR**	**Nomenclature in this paper**
Part 2	p91.67_ENMO_mg_0-24h_fullRecording	M120
Part 2	p95.83_ENMO_mg_0-24h_fullRecording	M60
Part 2	p97.92_ENMO_mg_0-24h_fullRecording	M30
Part 2	p98.96_ENMO_mg_0-24h_fullRecording	M15
Part 2	p99.31_ENMO_mg_0-24h_fullRecording	M10
Part 2	p99.65_ENMO_mg_0-24h_fullRecording	M5
Part 2	p99.86_ENMO_mg_0-24h_fullRecording	M2
Part 2	AD_mean_ENMO_mg_0-24hr	AvAcc
Part 2	AD_ig_gradient_ENMO_0-24hr	IG
Part 5	dur_day_total_LIG_min_pla	LPA
Part 5	dur_day_total_MOD_min_pla	MVPA
Part 5	dur_day_total_IN_min_pla	ST

Abbreviations: M120, the most active 120 minutes of the day; M60, the most active 60 minutes of the day; M30, the most active 30 minutes of the day; M15, the most active 15 minutes of the day; M10, the most active 10 minutes of the day; M5, the most active 5 minutes of the day; M2; the most active 2 minutes of the day; AvAcc, average acceleration; IG, intensity gradient; LPA, light physical activity; MVPA, moderate-to-vigorous physical activity; ST, sedentary time

Supplementary Table S2. Correlation matrix for the research model.

	Sex	Pos Affect	ST	LPA	MVPA	IG	AvACC	M120	M60	M30	M15	M10	M5	M2
Sex	1													
Pos Affect	-0.005	1												
ST	0.037	0.028	1											
LPA	0.018	0.013	**-0.351****	1										
MVPA	0.108	0.105	**-0.559****	**0.579****	1									
IG	0.062	**0.443****	-0.158	-0.113	**0.272***	1								
AvACC	0.102	0.238	**-0.528****	**0.584****	**0.896****	**0.450****	1							
M120	0.099	**0.275***	**-0.557****	**0.406****	**0.796****	**0.513****	**0.904****	1						
M60	0.175	**0.353****	**-0.498****	**0.311***	**0.702****	**0.598****	**0.847****	**0.955****	1					
M30	0.204	**0.406****	**-0.461****	**0.245**	**0.634****	**0.644****	**0.779****	**0.888****	**0.980****	1				
M15	0.226	**0.398****	**-0.436****	**0.196**	**0.562****	**0.694****	**0.712****	**0.812****	**0.928****	**0.979****	1			
M10	0.251	**0.344****	**-0.412****	**0.165**	**0.495****	**0.706****	**0.653****	**0.750****	**0.872****	**0.930****	**0.976****	1		
M5	0.255	**0.344****	**-0.383****	**0.145**	**0.417****	**0.702****	**0.577****	**0.674****	**0.804****	**0.876****	**0.941****	**0.982****	1	
M2	**0.263***	0.224	**-0.331***	**0.101**	**0.301***	**0.627****	**0.444****	**0.538****	**0.657****	**0.727****	**0.809****	**0.912****	**0.944****	1
FP2-FP1	**-0.266***	0.024	-0.054	-0.207	**-0.277***	-0.001	-0.145	-0.082	-0.010	0.026	0.044	0.041	0.066	0.031
AF4-AF3	-0.106	0.199	-0.078	-0.146	-0.183	0.079	-0.060	-0.057	0.003	0.039	0.070	0.101	0.126	0.134
F8-F7	**-0.270***	0.058	-0.066	-0.209	-0.216	0.110	-0.167	-0.146	-0.071	-0.025	0.009	0.022	0.041	0.036
F6-F5	-0.134	0.184	-0.002	-0.057	-0.025	0.154	0.001	0.017	0.099	0.145	0.173	0.179	0.180	0.160
F4-F3	-0.175	**0.310***	0.099	-0.226	-0.160	0.124	-0.118	-0.080	0.003	0.064	0.091	0.093	0.093	0.076
F2-F1	-0.117	**0.305***	0.082	-0.166	-0.172	0.045	-0.148	-0.108	-0.030	0.037	0.076	0.081	0.091	0.077
FT8-FT7	-0.062	-0.004	-0.173	-0.088	-0.062	0.100	-0.048	-0.074	0.009	0.055	0.096	0.128	0.145	0.174
P8-P7	0.004	-0.187	-0.228	0.171	0.101	0.062	0.150	0.028	0.060	0.060	0.073	0.077	0.079	0.050
P6-P5	-0.017	**-0.279***	-0.227	0.167	0.181	0.087	0.169	0.131	0.167	0.167	0.169	0.176	0.162	0.139
P4-P3	-0.059	-0.224	-0.132	0.134	0.067	-0.136	0.013	0.030	0.124	0.151	0.152	0.161	0.145	0.141
P2-P1	-0.134	**-0.268***	0.081	0.015	-0.093	**-0.368****	-0.106	-0.138	-0.133	-0.150	-0.166	-0.147	-0.166	-0.134

Abbreviations: Pos affect, Positive Affect; ST, sedentary time; LPA, light physical activity; MVPA, moderate-to-vigorous physical activity; IG, intensity gradient; AvAcc, average acceleration; M120, the most active 120 minutes of the day; M60, the most active 60 minutes of the day; M30, the most active 30 minutes of the day; M15, the most active 15 minutes of the day; M10, the most active 10 minutes of the day; M5, the most active 5 minutes of the day; M2; the most active 2 minutes of the day. Bolded electrodes are statistically significant. $^{*}p < 0.05$, $^{**}p < 0.01$

Supplementary Table S3. Regression model summary of AA power predicting ST accounting for positive affect and sex

Overall model for sex, affect, and EEG				Predictor					
	R^2	F	*p*	B	B_{error}	β	t	*p*	CI
FP2-FP1	0.004	0.079	0.971	-74.784	217.322	-0.048	-0.344	0.732	-510.307, 360.738
AF4-AF3	0.009	0.163	0.921	-119.281	196.581	-0.084	-0.607	0.546	-513.237, 274.676
F8-F7	0.006	0.106	0.956	-43.145	96.923	-0.062	-0.445	0.658	-237.383, 151.094
F6-F5	0.002	0.040	0.989	-2.154	130.170	-0.002	-0.017	0.987	-263.021, 258.712
F4-F3	0.013	0.238	0.870	119.639	155.326	0.110	0.770	0.444	-191.642, 430.921
F2-F1	0.009	0.165	0.920	150.162	245.331	0.087	0.612	0.543	-341.494, 641.819
FT8-FT7	0.031	0.594	0.622	-119.847	93.043	-0.171	-1.288	0.203	-306.310, 66.615
P8-P7	0.053	1.034	0.385	-79.774	46.245	-0.230	-1.725	0.090	-172.451, 12.902
P6-P5	0.054	1.042	0.381	-88.438	51.053	-0.237	-1.732	0.089	-190.750, 13.875
P4-P3	0.018	0.341	0.796	-69.501	73.156	-0.130	-0.950	0.346	-216.108, 77.106
P2-P1	0.012	0.218	0.884	93.316	127.722	0.103	0.731	0.468	-162.645, 349.277

Abbreviations: R^2, R square, B, unstandardized beta coefficients, B_{error}, unstandardized standard error, CI, Confidence interval 95%.

Supplementary Table S4. Regression model summary of AA power predicting LPA accounting for positive affect and sex

Overall model for sex, affect, and EEG				Predictor					
	R^2	F	*p*	B	B_{error}	β	t	*p*	CI
FP2-FP1	0.045	0.856	0.469	-125.077	78.481	-0.218	-1.594	0.117	-282.357, 32.202
AF4-AF3	0.023	0.436	0.728	-81.382	71.944	-0.155	-1.131	0.263	-225.560, 62.797
F8-F7	0.046	0.886	0.454	-56.743	35.000	-0.222	-1.621	0.111	-126.885, 13.399
F6-F5	0.004	0.072	0.975	-20.783	47.948	-0.060	-0.433	0.666	-116.873, 75.306
F4-F3	0.059	1.155	0.335	-103.625	55.900	-0.259	-1.854	0.069	-215.651, 8.401
F2-F1	0.032	0.611	0.611	-120.002	89.376	-0.188	-1.343	0.185	-299.117, 59.112
FT8-FT7	0.008	0.149	0.930	-22.491	34.712	-0.087	-0.648	0.520	-92.056, 47.074
P8-P7	0.032	0.599	0.619	22.920	17.244	0.180	1.329	0.189	-11.637, 57.478
P6-P5	0.032	0.614	0.609	25.636	19.033	0.186	1.347	0.184	-12.507, 63.779
P4-P3	0.021	0.387	0.763	28.670	26.937	0.146	1.064	0.292	-25.314, 82.653
P2-P1	0.001	0.018	0.997	7.819	47.343	0.023	0.165	0.869	-87.059, 102.696

Abbreviations: R^2, R square, B, unstandardized beta coefficients, B_{error}, unstandardized standard error, CI, Confidence interval 95%.

Supplementary Table S5. Regression model summary of AA power predicting MVPA accounting for positive affect and sex

Overall model for sex, affect, and EEG				Predictor					
	R^2	F	*p*	B	B_{error}	β	t	*p*	CI
FP2-FP1	0.090	1.822	0.154	-159.825	79.096	-0.270	-2.021	0.048	-318.337, -1.314
AF4-AF3	0.062	1.208	0.315	-110.087	72.825	-0.203	-1.512	0.136	-256.033, 35.858
F8-F7	0.063	1.236	0.306	-55.097	35.826	-0.209	-1.538	0.130	-126.894, 16.700
F6-F5	0.024	0.447	0.720	-11.300	49.027	-0.032	-0.230	0.819	-109.552, 86.952
F4-F3	0.057	1.116	0.351	-82.010	57.796	-0.199	-1.419	0.162	-197.837, 33.817
F2-F1	0.064	1.251	0.300	-140.957	90.792	-0.214	-1.552	0.126	-322.904, 40.999
FT8-FT7	0.026	0.486	0.693	-14.570	35.531	-0.055	-0.410	0.683	-85.776, 56.636
P8-P7	0.038	0.720	0.544	16.417	17.754	0.125	0.925	0.359	-19.162, 51.996
P6-P5	0.072	1.414	0.249	32.721	19.257	0.230	1.699	0.095	-5.870, 71.312
P4-P3	0.033	0.623	0.603	20.879	27.649	0.103	0.755	0.453	-34.530, 76.289
P2-P1	0.026	0.482	0.696	-19.128	48.292	-0.055	-0.396	0.694	-115.908, 77.652

Abbreviations: R^2, R square, B, unstandardized beta coefficients, B_{error}, unstandardized standard error, CI, Confidence interval 95%.

Supplementary Table S6. Regression model summary of AA power predicting AvACC accounting for positive affect and sex

Overall model for sex, affect, and EEG				Predictor					
	R^2	F	*p*	B	B_{error}	β	t	*p*	CI
FP2-FP1	0.083	1.670	0.184	-14.899	15.076	-0.132	-0.988	0.327	-45.113, 15.315
AF4-AF3	0.077	1.532	0.217	-10.527	13.716	-0.102	-0.768	0.446	-38.014, 16.960
F8-F7	0.093	1.869	0.146	-8.289	6.696	-0.165	-1.238	0.221	-21.707, 5.130
F6-F5	0.068	1.340	0.271	-2.066	9.096	-0.030	-0.227	0.821	-20.295, 16.163
F4-F3	0.102	2.077	0.114	-15.583	10.713	-0.199	-1.455	0.151	-37.053, 5.887
F2-F1	0.116	2.408	0.077	-29.212	16.753	-0.234	-1.744	0.087	-62.786, 4.362
FT8-FT7	0.069	1.356	0.266	-2.061	6.596	-0.041	-0.313	0.756	-15.281, 11.158
P8-P7	0.106	2.179	0.101	5.035	3.249	0.201	1.550	0.127	-1.476, 11.547
P6-P5	0.128	2.702	0.054	6.965	3.543	0.258	1.966	0.054	-0.135, 14.066
P4-P3	0.073	1.438	0.242	2.945	5.141	0.076	0.573	0.569	-7.358, 13.247
P2-P1	0.068	1.340	0.271	-2.040	8.968	-0.031	-0.227	0.821	-20.013, 15.933

Abbreviations: R^2, R square, B, unstandardized beta coefficients, B_{error}, unstandardized standard error, CI, Confidence interval 95%.

Supplementary Table S7. Regression model summary of AA power predicting M120 accounting for positive affect and sex

Overall model for sex, affect, and EEG				Predictor					
	R^2	F	*p*	B	B_{error}	β	t	*p*	CI
FP2-FP1	0.090	1.808	0.156	-22.946	46.186	-0.066	-0.497	0.621	-115.505, 69.614
AF4-AF3	0.096	1.958	0.131	-33.802	41.719	-0.107	-0.810	0.421	-117.408, 49.803
F8-F7	0.105	2.160	0.103	-22.502	20.436	-0.146	-1.101	0.276	-63.456, 18.453
F6-F5	0.086	1.728	0.172	-4.498	27.690	-0.022	-0.162	0.872	-59.990, 50.994
F4-F3	0.111	2.280	0.090	-40.662	32.772	-0.169	-1.241	0.220	-106.337, 25.014
F2-F1	0.122	2.547	0.065	-77.372	51.328	-0.201	-1.507	0.137	-180.236, 25.491
FT8-FT7	0.090	1.817	0.155	-10.414	20.044	-0.067	-0.520	0.605	-50.583, 29.756
P8-P7	0.092	1.863	0.147	6.338	10.066	0.082	0.630	0.532	-13.835, 26.511
P6-P5	0.133	2.822	0.047	18.897	10.860	0.227	1.740	0.087	-2.867, 40.662
P4-P3	0.096	1.942	0.134	12.223	15.606	0.103	0.783	0.437	-19.052, 43.498
P2-P1	0.089	1.781	0.162	-11.275	27.265	-0.056	-0.414	0.681	-65.915, 43.366

Abbreviations: R^2, R square, B, unstandardized beta coefficients, B_{error}, unstandardized standard error, CI, Confidence interval 95%.

Supplementary Table S8. Regression model summary of AA power predicting M2 accounting for positive affect and sex

Overall model for sex, affect, and EEG				Predictor					
	R^2	F	*p*	B	B_{error}	β	t	*p*	CI
FP2-FP1	0.130	2.732	0.052	229.012	289.813	0.103	0.790	0.433	-351.786, 809.810
AF4-AF3	0.134	2.844	0.046	251.181	262.054	0.123	0.959	0.342	-273.988, 776.349
F8-F7	0.130	2.728	0.053	101.224	129.361	0.102	0.782	0.437	-158.021, 360.470
F6-F5	0.145	3.103	0.034	217.751	171.896	0.162	1.267	0.211	-126.737, 562.238
F4-F3	0.123	2.572	0.063	93.486	208.826	0.060	0.448	0.656	-325.011, 511.983
F2-F1	0.122	2.537	0.066	109.193	329.454	0.044	0.331	0.742	-551.047, 769.433
FT8-FT7	0.157	3.402	0.024	191.557	123.849	0.192	1.547	0.128	-56.641, 439.756
P8-P7	0.128	2.702	0.054	46.697	63.294	0.095	0.738	0.464	-80.147, 173.541
P6-P5	0.166	3.647	0.018	119.184	68.372	0.224	1.743	0.087	-17.837, 256.205
P4-P3	0.165	3.623	0.019	166.029	96.236	0.219	1.725	0.090	-26.832, 358.890
P2-P1	0.121	2.533	0.066	-54.225	171.779	-0.042	-0.316	0.753	-398.478, 290.027

Abbreviations: R^2, R square, B, unstandardized beta coefficients, B_{error}, unstandardized standard error, CI, Confidence interval 95%.

Supplementary Table S9. Regression model summary of AA power predicting IG accounting for positive affect and sex

	Model			Predictor					
	R^2	F	*p*	B	B_{error}	β	t	*p*	CI
FP2-FP1	0.200	4.593	0.006	0.016	0.327	0.006	0.050	0.960	-0.639, 0.672
AF4-AF3	0.200	4.592	0.006	-0.006	0.296	-0.002	-0.019	0.985	-0.600, 0.588
F8-F7	0.211	4.915	0.004	0.128	0.145	0.110	0.880	0.382	-0.163, 0.418
F6-F5	0.207	4.792	0.005	0.135	0.195	0.085	0.693	0.491	-0.26, 0.525
F4-F3	0.200	4.592	0.006	-0.004	0.235	-0.002	-0.018	0.986	-0.474, 0.466
F2-F1	0.208	4.815	0.005	-0.270	0.368	-0.093	-0.732	0.467	-1.007, 0.468
FT8-FT7	0.211	4.917	0.004	0.124	0.141	0.106	0.883	0.381	-0.158, 0.407
P8-P7	0.222	5.233	0.003	0.087	0.070	0.150	1.240	0.220	-0.054, 0.228
P6-P5	0.249	6.083	0.001	0.144	0.076	0.230	1.892	0.064	-0.009, 0.297
P4-P3	0.201	4.625	0.006	-0.031	0.111	-0.035	-0.281	0.780	-0.253, 0.191
**P2-P1**	0.264	6.573	0.001	-0.403	0.185	-0.264	-2.180	**0.034***	-0.774, -0.033

Abbreviations: R^2, R square, B, unstandardized beta coefficients, B_{error}, unstandardized standard error, CI, Confidence interval 95%. Bolded electrodes are statistically significant. *$p < 0.05$.

Supplementary Table S10. Regression model summary of AA power predicting M60 accounting for positive affect and sex

	Model			Predictor					
	R^2	F	*p*	B	B_{error}	β	t	*p*	CI
FP2-FP1	0.157	3.406	0.024	15.112	63.589	0.031	0.238	0.813	-112.324, 142.547
AF4-AF3	0.158	3.448	0.023	-23.271	57.595	-0.051	-0.404	0.688	-138.693, 92.151
F8-F7	0.158	3.438	0.023	-10.571	28.359	-0.048	-0.373	0.711	-67.404, 46.263
F6-F5	0.159	3.472	0.022	18.032	37.989	0.060	0.475	0.637	-58.100, 94.164
F4-F3	0.162	3.555	0.020	-29.963	45.489	-0.087	-0.659	0.513	-121.124, 61.199
F2-F1	0.171	3.783	0.015	-71.809	71.335	-0.131	-1.007	0.319	-214.767, 71.149
FT8-FT7	0.156	3.395	0.024	4.688	27.610	0.021	0.170	0.866	-50.643, 60.019
P8-P7	0.172	3.808	0.015	14.258	13.751	0.130	1.037	0.304	-13.300, 41.815
**P6-P5**	0.234	5.614	0.002	34.700	14.600	0.292	2.377	**0.021***	5.441, 63.960
P4-P3	0.204	4.701	0.005	38.259	20.942	0.226	1.827	0.073	-3.711, 80.228
P2-P1	0.156	3.390	0.024	-4.707	37.526	-0.016	-0.125	0.901	-79.912, 70.498

Abbreviations: R^2, R square, B, unstandardized beta coefficients, B_{error}, unstandardized standard error, CI, Confidence interval 95%. Bolded electrodes are statistically significant. *$p < 0.05$.

Supplementary Table S11. Regression model summary of AA power predicting M30 accounting for positive affect and sex

	Model			Predictor					
	R^2	F	*p*	B	B_{error}	β	t	*p*	CI
FP2-FP1	0.213	4.959	0.004	50.831	82.094	0.077	0.619	0.538	-113.688, 215.351
AF4-AF3	0.208	4.810	0.005	-12.712	74.666	-0.021	-0.170	0.865	-162.347, 136.922
F8-F7	0.207	4.800	0.005	2.238	36.766	0.008	0.061	0.952	-71.442, 75.918
F6-F5	0.217	5.095	0.003	41.140	48.977	0.103	0.840	0.405	-57.012, 139.292
F4-F3	0.208	4.820	0.005	-13.609	59.104	-0.030	-0.230	0.819	-132.055, 104.838
F2-F1	0.212	4.926	0.004	-51.319	92.956	-0.070	-0.552	0.583	-237.607, 134.969
FT8-FT7	0.212	4.939	0.004	20.636	35.651	0.069	0.579	0.565	-50.810, 92.081
P8-P7	0.226	5.367	0.003	20.656	17.761	0.140	1.163	0.250	-14.939, 56.250
**P6-P5**	0.295	7.669	0.000	48.924	18.724	0.308	2.613	**0.012***	11.401, 86.448
**P4-P3**	0.276	6.976	0.000	60.759	26.699	0.268	2.276	**0.027***	7.254, 114.264
P2-P1	0.208	4.803	0.005	-5.492	48.592	-0.014	-0.113	0.910	-102.871, 91.888

Abbreviations: R^2, R square, B, unstandardized beta coefficients, B_{error}, unstandardized standard error, CI, Confidence interval 95%. Bolded electrodes are statistically significant. *p < 0.05.

Supplementary Table S12. Regression model summary of AA power predicting M15 accounting for positive affect and sex

	Model			Predictor					
	R^2	F	*p*	B	B_{error}	β	t	*p*	CI
FP2-FP1	0.220	5.164	0.003	86.902	104.988	0.102	0.828	0.411	-123.497, 297.302
AF4-AF3	0.210	4.882	0.004	11.823	95.760	0.015	0.123	0.902	-180.085, 203.731
F8-F7	0.212	4.946	0.004	19.240	47.077	0.051	0.409	0.684	-75.104, 113.584
F6-F5	0.228	5.414	0.002	70.568	62.487	0.137	1.129	0.264	-54.659, 195.795
F4-F3	0.210	4.877	0.004	4.885	75.825	0.008	0.064	0.949	-147.072, 156.843
F2-F1	0.210	4.887	0.004	-19.675	119.503	-0.021	-0.165	0.870	-259.163, 219.814
FT8-FT7	0.223	5.250	0.003	42.881	45.490	0.112	0.943	0.350	-48.283, 134.044
P8-P7	0.232	5.547	0.002	28.678	22.728	0.152	1.262	0.212	-16.870, 74.226
**P6-P5**	0.297	7.761	0.000	62.783	24.009	0.308	2.615	**0.011***	14.668, 110.897
**P4-P3**	0.278	7.076	0.000	78.156	34.227	0.269	2.283	**0.026***	9.563, 146.748
P2-P1	0.211	4.902	0.004	-15.583	62.283	-0.031	-0.250	0.803	-140.402, 109.236

Abbreviations: R^2, R square, B, unstandardized beta coefficients, B_{error}, unstandardized standard error, CI, Confidence interval 95%.Bolded electrodes are statistically significant. *p < 0.05.

Supplementary Table S13. Regression model summary of AA power predicting M10 accounting for positive affect and sex

	Model			Predictor					
	R^2	F	*p*	B	B_{error}	β	t	*p*	CI
FP2-FP1	0.193	4.379	0.008	110.747	129.057	0.108	0.858	0.395	-147.889, 369.382
AF4-AF3	0.186	4.179	0.010	58.301	117.522	0.062	0.496	0.622	-177.218, 293.820
F8-F7	0.187	4.227	0.009	34.854	57.793	0.076	0.603	0.549	-80.966, 150.674
F6-F5	0.205	4.737	0.005	97.398	76.616	0.157	1.271	0.209	-56.145, 250.940
F4-F3	0.183	4.107	0.011	24.631	93.196	0.034	0.264	0.793	-162.139, 211.400
F2-F1	0.182	4.079	0.011	6.426	147.001	0.006	0.044	0.965	-288.170, 301.022
FT8-FT7	0.203	4.674	0.006	67.289	55.660	0.146	1.209	0.232	-44.255, 178.834
P8-P7	0.202	4.654	0.006	33.271	27.996	0.146	1.188	0.240	-22.835, 89.376
**P6-P5**	0.265	6.606	0.001	73.913	29.679	0.300	2.490	**0.016***	14.434, 133.391
**P4-P3**	0.250	6.105	0.001	94.063	42.178	0.268	2.230	**0.030***	9.538, 178.589
P2-P1	0.182	4.091	0.011	-13.272	76.620	-0.022	-0.173	0.863	-166.823, 140.278

Abbreviations: R^2, R square, B, unstandardized beta coefficients, B_{error}, unstandardized standard error, CI, Confidence interval 95%. Bolded electrodes are statistically significant. *p < 0.05.

Supplementary Table S14. Regression model summary of AA power predicting M5 accounting for positive affect and sex

	Model			Predictor					
	R^2	F	*p*	B	B_{error}	β	t	*p*	CI
FP2-FP1	0.202	4.635	0.006	180.002	169.957	0.136	1.085	0.283	-152.583, 512.586
AF4-AF3	0.192	4.360	0.008	107.507	151.366	0.088	0.710	0.481	-195.836, 410.850
F8-F7	0.194	4.400	0.008	57.734	74.450	0.098	0.775	0.441	-91.468, 206.936
F6-F5	0.209	4.837	0.005	127.804	98.863	0.159	1.293	0.202	-70.321, 325.929
F4-F3	0.186	4.183	0.010	32.145	120.313	0.035	0.267	0.790	-208.968, 273.259
F2-F1	0.185	4.162	0.010	26.303	189.746	0.018	0.139	0.890	-353.957, 406.562
FT8-FT7	0.211	4.903	0.004	96.973	71.621	0.162	1.354	0.181	-46.558, 240.504
P8-P7	0.206	4.748	0.005	43.550	36.129	0.147	1.205	0.233	-28.855, 115.955
**P6-P5**	0.260	6.440	0.001	91.054	38.508	0.286	2.365	**0.022***	13.882, 168.227
**P4-P3**	0.244	5.918	0.001	113.726	54.752	0.250	2.077	**0.042***	4.000, 223.453
P2-P1	0.186	4.200	0.010	-33.416	98.840	-0.043	-0.338	0.737	-231.496, 164.664

Abbreviations: R^2, R square, B, unstandardized beta coefficients, B_{error}, unstandardized standard error, CI, Confidence interval 95%. Bolded electrodes are statistically significant. *p < 0.05.
